# Designing Emails Aimed at Increasing Family Physicians’ Use of a Web-Based Audit and Feedback Tool to Improve Cancer Screening Rates: Cocreation Process

**DOI:** 10.2196/humanfactors.9875

**Published:** 2018-09-04

**Authors:** Caroline A Bravo, Diego Llovet, Holly O Witteman, Laura Desveaux, Justin Presseau, Marianne Saragosa, Gratianne Vaisson, Shama Umar, Jill Tinmouth, Noah M Ivers

**Affiliations:** ^1^ Prevention and Cancer Control Cancer Care Ontario Toronto, ON Canada; ^2^ Institute of Health Policy, Management, and Evaluation University of Toronto Toronto, ON Canada; ^3^ Department of Family and Emergency Medicine Faculty of Medicine Laval University Quebec City, QC Canada; ^4^ Office of Education and Professional Development Faculty of Medicine Laval University Quebec City, QC Canada; ^5^ Research Centre of the CHU de Québec Quebec City, QC Canada; ^6^ Women’s College Research Institute Women’s College Hospital Toronto, ON Canada; ^7^ Institute for Health System Solutions and Virtual Care Women's College Hospital Toronto, ON Canada; ^8^ Clinical Epidemiology Program Ottawa Hospital Research Institute Ottawa, ON Canada; ^9^ School of Epidemiology and Public Health Faculty of Medicine University of Ottawa Ottawa, ON Canada; ^10^ School of Psychology Faculty of Social Sciences University of Ottawa Ottawa, ON Canada; ^11^ Family Practice Health Centre Women's College Hospital Toronto, ON Canada; ^12^ Institute for Clinical Evaluative Sciences Toronto, ON Canada; ^13^ Department of Medicine Faculty of Medicine University of Toronto Toronto, ON Canada; ^14^ Sunnybrook Research Institute Toronto, ON Canada; ^15^ Department of Family and Community Medicine Faculty of Medicine University of Toronto Toronto, ON Canada

**Keywords:** audit and feedback, clinical audit, co-creation, user design, email, behavior change, physician engagement

## Abstract

**Background:**

Providing clinical performance data to health professionals, a process known as audit and feedback, can play an important role in health system improvement. However, audit and feedback tools can only be effective if the targeted health professionals access and actively review their data. Email is used by Cancer Care Ontario, a provincial cancer agency, to promote access to a Web-based audit and feedback tool called the Screening Activity Report (SAR); however, current emails that lack behavior change content have been ineffective at encouraging log-in to the SAR.

**Objective:**

The objective of our study was to describe the process and experience of developing email content that incorporates user input and behavior change techniques (BCTs) to promote the use of the SAR among Ontario primary care providers.

**Methods:**

Our interdisciplinary research team first identified BCTs shown to be effective in other settings that could be adapted to promote use of the SAR. We then developed draft BCT-informed email content. Next, we conducted cocreation workshops with physicians who had logged in to the SAR more than once over the past year. Participants provided reactions to researcher-developed BCT-informed content and helped to develop an email that they believed would prompt their colleagues to use the SAR. Content from cocreation workshops was brought to focus groups with physicians who had not used the SAR in the past year. We analyzed notes from the cocreation workshops and focus groups to inform decisions about content. Finally, 8 emails were created to test BCT-informed content in a 2×2×2 factorial randomized experiment.

**Results:**

We identified 3 key tensions during the development of the email that required us to balance user input with scientific evidence, organizational policies, and our scientific objectives, which are as follows: conflict between user preference and scientific evidence, privacy constraints around personalizing unencrypted emails with performance data, and using cocreation methods in a study with the objective of developing an email that featured BCT-informed content.

**Conclusions:**

Teams tasked with developing content to promote health professional engagement with audit and feedback or other quality improvement tools might consider cocreation processes for developing communications that are informed by both users and BCTs. Teams should be cautious about making decisions solely based on user reactions because what users seem to prefer is not always the same as what works. Furthermore, implementing user recommendations may not always be feasible. Teams may face challenges when using cocreation methods to develop a product with the simultaneous goal of having clearly defined variables to test in later studies. The expected role of users, evidence, and the implementation context all warrant consideration to determine whether and how cocreation methods could help to achieve design and scientific objectives.

## Introduction

Audit and feedback is a widely adopted strategy to improve clinical practice guideline adherence [1]. However, audit and feedback tools can only be effective if physicians access them to review their clinical performance data. Physicians who do not see their performance feedback data are not likely to use it for quality improvement [2]. Email is commonly used to communicate with physicians and can be an effective channel for encouraging guideline-recommended care [3] and access to Web-based educational opportunities [4]. Cancer Care Ontario, a provincial government cancer agency, uses email communications to encourage primary care physicians to log in to a Web-based cancer screening tool called the Screening Activity Report (SAR).

The SAR is a Web-based audit and feedback tool aimed at improving cancer screening-guideline adherence among primary care physicians in Ontario, Canada. The SAR contains patient-level information about rostered patients (ie, patients that are enrolled with a physician) who require action regarding cancer screening (eg, due for screening, overdue for screening, and require follow-up to an abnormal result). To promote regular SAR use, Cancer Care Ontario sends monthly emails to family physicians to inform them that the SAR data has been updated. However, the content of these monthly emails appears to be ineffective at promoting access to the SAR; only 2.37% (129/5445), 3.76% (207/5512), and 4.09% (227/5552) of the SAR-registered physicians logged in to the SAR in February, March, and April 2017, respectively [5]. Additionally, in the same period, less than half of recipients opened the email and only about 7% clicked on the embedded link to access the SAR [6]. Thus, there is a need to understand how to develop emails that are more effective at encouraging family physicians to log in and use the audit and feedback tool.

There may be a number of reasons why physicians do not open the monthly SAR email, including feeling overwhelmed by the number of emails received and having limited time to open emails [7]. Another potential reason may be that physicians dismiss these emails owing to a lack of compelling content (eg, benefits to the physician and patient of using the SAR). It may be possible to improve SAR access with content that employs a behavioral science approach to address barriers to SAR use.

In this paper, we describe our process and experience of developing email content involving cocreation workshops and focus group discussions with physician SAR users. The goal of this process was to develop user- and behavior change technique (BCT)-informed email content for a study testing variants of the email [8]. Using BCT classification systems helps to build evidence about which behavior change intervention components work and under what conditions [9].

## Methods

### Design and Setting

We conducted cocreation workshops and focus groups with family physicians in Toronto and Kingston, Ontario, Canada between January and April 2017. Final email products were tested in a 2×2×2 factorial experiment with Ontario physicians registered for the SAR [8].

### Email Development Process

We used an iterative process to develop user-informed email content that operationalizes 3 BCTs. Textbox 1 illustrates the steps involved in our email content development process. Throughout the process, we engaged relevant decision makers at Cancer Care Ontario to review content and provide feedback to ensure that the final products would be implementable.

**Email content development process.**

Step 1. Interdisciplinary research team selects behavior change techniques (BCTs) and develops draft content.

- Reviewed existing literature on behavior change among physicians [10].
- Identified potential BCTs from Michie et al’s BCT taxonomy (v1) [11].
- Held a team creative writing session to develop sample messages inspired by the examples on Michie et al’s BCT taxonomy [11].

Outcome: Sample messages that operationalized BCTs to bring to cocreation workshops for critique, refinement, or replacement by participants.

Step 2. Recruit users.

- Used a combination of purposive [12] and convenience sampling [13] to recruit adopter and nonadopters for cocreation workshops and focus groups.
  - Adopter = physician user who had logged in to the SAR more than once within the 12 months prior to our recruitment period.
  - Nonadopter = physician users who had not logged in to the SAR within those 12 months.
  - Definitions were developed in collaboration with Cancer Care Ontario. Cancer screening for any given patient does not need to happen more than once per year; therefore, it is reasonable to check who is overdue annually. However, it is ideal to do so more often to minimize how far overdue a patient would be and to identify patients that require follow-up to an abnormal result. Additionally, many physicians access the SAR once a year to calculate their cancer screening rates for their application to earn an annual Preventive Care Bonus.
- Participants were offered a Can $200 gift card in appreciation of their time and effort.

Outcome: Nine adopter participants for cocreation workshops. Eleven participants (adopter and nonadopter) for focus groups. No participant took part in more than one cocreation workshop or focus group.

Step 3. Conduct cocreation workshops with adopters.

- Adapted interview guides for workshops from a planning and design tool for codesign workshops [14].
- Held one 2-hour workshop with adopter participants in Toronto, Ontario. Participants provided feedback on BCT-informed messages developed by the research team in step 1 and produced emails with content that they thought would convince their nonuser counterparts to log in to the SAR.
- Audio-recorded workshops and created detailed notes of reactions to content. Research team met to review adopter-generated content and considered the valence (positive or negative) of reactions to each BCT-informed message developed during step 1, the evidence of effectiveness of each BCT, and the potential risks of using messages given any negative reactions.
- Research team developed 2 emails that combined user-generated content and researcher-developed BCT-informed messages to bring to the second workshop.
- Held one 2-hour workshop with adopter participants in Kingston, Ontario. Participants provided feedback on both emails and refined as needed.
- Research team met to review detailed notes from audio recordings, analyze content for underlying BCTs, and review the valence of reactions.

Outcome: Adopter-generated content and feedback to sample messages.

Step 4. Pretest content with adopters and nonadopters.

- Held three 2-hour focus groups in Toronto and Kingston, Ontario with different adopter and nonadopter participants in each.
- Sent emails that were developed and refined by adopters and the research team in step 3 to participants. Participants viewed emails on their personal cell phone during the focus group. Participants provided reactions and refined content as needed.
- Following each session, the research team met to review detailed notes from audio recordings and discuss user-generated content and participant feedback including verbal reactions to the materials and recommendations for content changes. Considerations for content changes included scientific evidence of effectiveness for each BCT, strength and frequency of the reaction (eg, severity of sentiment such as strong distaste or strong liking, number of participants that shared similar reactions), and feasibility of implementing the recommendation.

Outcome: Adopter-generated content and feedback to emails produced in step 3.

Step 5. Finalize products (emails) for testing.

- Developed 8 different email versions using the user-generated and BCT-informed content that was refined and finalized by the end of step 4.
- Variants of email differed by the number of BCTs operationalized, resulting in different word counts and length.

Outcome: Eight email variants for testing 3 BCTs in a 2×2×2 factorial randomized experiment [8].

## Results

### Participants

Although we made an effort to recruit adopters for cocreation workshops and nonadopter participants for focus groups, challenges with recruitment required us to be open to the inclusion of adopter participants in the focus groups. Table 1 and Table 2 show the participant characteristics for cocreation workshops and focus groups.

### Behavior Change Techniques

The full list of BCTs that were initially selected by the research team and considered throughout the project can be found in Multimedia Appendix 1.

The 3 BCTs to be tested as part of the 2×2×2 factorial randomized experiment include the following: anticipated regret, material incentive (behavior), and problem solving. The final operationalizations of each BCT can be found in Multimedia Appendix 2. The final email with all 3 BCTs can be found in the Multimedia Appendix 3.

### Key Tensions and Resulting Changes to Email Content

Over the course of the email development process, we used 3 main sources of input to inform decisions about content, including scientific literature, SAR users, and Cancer Care Ontario communication policies. Tensions arose when these sources of input conflicted with each other.

#### Conflict Between User Preferences and Scientific Evidence

Given the evidence that physicians’ decisions are influenced by the desire to minimize regret associated with a potentially wrong decision [15-17], we developed anticipated regret content and rendered it in the form of a reflective question. At first, we considered missing an abnormal result to be the outcome of regret (ie, “How would you feel if you missed an abnormal result?”). However, after discussion with adopters, we refined the anticipated regret content to more explicitly focus on the effect of missing an abnormal test result on patient outcomes (ie, “How would you feel if a patient had a poor outcome because you missed an abnormal test result?”).

Tensions emerged when users’ reactions conflicted with evidence about the effectiveness of the anticipated regret. Focus group participants expressed feelings of guilt and anger when they read the message and indicated that they did not want to have an emotional response when reading an email. Focus group participants described the anticipated regret content as “confrontational,” “combative,” and “not a nice way of starting.”

**Table 1:** Participant characteristics for cocreation workshops.

Characteristic		All cocreation workshops (n=9)	Cocreation workshop #1 in Toronto, Ontario (n=7)	Cocreation workshop #2 in Kingston, Ontario (n=2)
**Gender, n**				
	Male	2	1	1
	Female	7	6	1
**Type of user, n**				
	Adopter	9	7	2
	Nonadopter	0	0	0
**Years practicing family medicine**				
	Range	3-32	3-32	12-30
	Average number of years	15	13	21

**Table 2:** Participant characteristics for focus groups.

Characteristic		Total for all focus groups (n=11)	Focus group #1 in Toronto, Ontario (n=3)	Focus group #2 in Kingston, Ontario (n=3)	Focus group #3 in Toronto, Ontario (n=5)
**Gender, n**					
	Male	6	2	1	3
	Female	5	1	2	2
**Type of user, n**					
	Adopter	4	1	0	2
	Nonadopter	7	2	3	3
**Years practicing family medicine**					
	Range	4-32	4-27	18-30	5-32
	Average number of years	20	19	24	19

One participant restated this message as:

How would you feel if you were a murderer? How would you feel if you screwed up?

SAR Non adopter P019

Further, participants articulated that the content implied that they were not doing their job well. For example, one participant stated:

You know it happens, but you don’t have to sort of start off a positive report that says “we would really like...we think this report is really encouraging you to use it, it can help you with A, B, and C, but you know what really if you missed a test result you’re shitty, you’re really lousy, you’re going to be sued, so use this tool.”...that’s just so condescending, negative on...you know the stress in our life is already there, we don’t need to be told that we feel bad when things go bad.

SAR Non adopter P016

Exploration of reactions in focus groups revealed that the content, though evoking negative emotions, motivated several participants to read the email further; for example, one participant said:

I would read more but I would be pissed off.

SAR Non adopter P017

Another participant acknowledged that the guilt was motivating, aligning with evidence behind the BCT:

I think the first line is good, how would you feel if patient had a poor outcome because you missed an abnormal test result? I think that’s every doctor’s fear because we go through so many results and we’re making decisions very quickly and it is possible to miss test results, which everybody’s done. And sometimes you get lucky and it’s not a big deal, and sometimes you don’t and you feel horrible about it so I think the guilt thing in there is good, and it’s motivating.

SAR Non adopter P011

Thus, although we recognized that too much fear can result in physicians avoiding the desired behavior (ie, logging in to the SAR), we believed that these strong negative reactions had the potential to engage recipients and maintain their attention, something that the previous SAR email was seemingly unable to do. We tried to avoid a judgmental tone that would evoke anger and to emphasize in the content that followed the anticipated regret message that logging in to the SAR could mitigate feelings of fear and anxiety about missing critical cancer screening results.

Participants also had negative reactions to the embedded problem-solving strategies for making time to access the SAR. The problem-solving BCT requires analysis of factors influencing the behavior and selecting strategies that overcome barriers or increase facilitators [11]. Our problem-solving strategies for improved SAR use were informed directly by adopters’ real-world experiences, which included assigning a delegate to access the SAR on their behalf to overcome time pressures. One adopter described why nonadopters such as her colleagues need problem-solving strategies:

Because it dumbs [accessing the SAR] down, it makes it a stepwise option for them to do. It’s perfect. It’s perfect. That’s what they need. They just need someone to take them by the hand and say, ‘Brian, print this out and give it to Dale and she will book you 15 minutes.’ They can’t find 15 minutes in their life to do this so this is a recipe for following through. It’s really good. I like it a lot.

SAR Adopter P008

Nevertheless, several focus group participants expressed that they would not use these strategies, often stating that resource and time constraints prevented them from employing them in their practice. In some cases, participants disliked the strategies proposed because they felt that they were unnecessary; for example, one participant said that if they are going to do any clicking, they might as well click into the SAR rather than click into their calendar to make a reminder about logging into the SAR. Moreover, focus group participants described this strategy to book time in their calendar as “patronizing,” “trivial,” and “petty.” For example, one participant stated:

I think some of the tips are unnecessary. Like, book some calendar time. I know I can do that. I know it’s one of those things everyone should fit in their schedule; however, they can’t. So telling me to put it in your calendar, I’m a grown up.

SAR Non adopter P018

Though our goal was to provide strategies that helped users overcome time and workload challenges, participants often wanted to eliminate the BCT-informed content and recommended that we “just get to the bottom line*-* log in to the SAR.”

#### Privacy Constraints Around Personalizing Unencrypted Emails With Performance Data

One significant source of tension occurred when organizational privacy constraints restricted our ability to respond to some content and design changes offered by participants, especially in the case of personalizing content using individualized performance data and embedded performance comparator data.

When prompted with the following message “10,000 women in Ontario need follow-up to an abnormal pap test; is one of them yours?,” focus group participants wanted the statement to be more personal and reflect the number of their patients that require follow-up:

Send me an email saying I got 7 abnormal and give me a link to log in. You don’t have to give me patient demographics and names or whatever. Or you can say, ‘we’ve identified patients based on whatever you know, please log in to this to get your confidential list’...you’re sending out an email that you want us to sort of attract physicians, you want to sort of catch them, you want to say. ‘Hey listen, we’ve identified all these abnormal results, they’ve been sitting here in our whatever database.’…notify me that I have 8 patients, that I guarantee you would motivate me to open up and then follow-up but...

SAR Non adopter P019

Yeah, the fact that I got an abnormal it’s helpful for me to know. You’re helping me because you’re actually letting me know that there’s some abnormals you want to make sure that I am aware of them and you want to make sure it’s like you feel as if your part of a team, ‘Hey we’re sending you this as a reminder that you have some abnormals.’ You sort of feel like it’s not the stick, and the carrot is that you’re actually improving on patient care.

SAR Non adopter P017

Though participants wanted varying degrees of personalized data (eg, Ontario rates vs individualized follow-up rates), it was clear that personalized data regarding the number of their own patients that required follow-up resonated with participants and had the potential to prompt SAR access.

In addition to personalized data, participants expressed a desire to have performance comparator data. Adopters described performance comparator information as “definitely the most unique thing” about the SAR in comparison to other tools used to monitor cancer screening in their practice, such as electronic medical records. One adopter expressed feeling comfort in numbers:

Because you have no sense of [how] the rest of the family physicians in Ontario are functioning...you really do function in a bubble.

SAR Adopter P008

Similarly, focus group participants found this benefit of the SAR “very interesting” and noted that it sparked curiosity. One participant stated that they were interested in the comparison data from both a medical and legal standpoint:

...I am interested in that if I have a glaring difference from other practices, I want to know that from both a medical and a legal point of view.

SAR Non adopter P013

Although most focus group participants agreed that comparison information was useful, they also reported that the content used to communicate this feature of the SAR was not as compelling as it could be. One participant suggested that the comparator data would be a strong motivator to use the SAR:

If the data was planted right in the email. Like, this is you, this is people in the LHIN, and this is people in Ontario.

SAR Non adopter P012

Furthermore, focus group participants thought an image displaying their performance data would motivate them to log in to the SAR, especially if the image showed that they were underperforming in comparison to their peers:

If there was an image there that I had a gauge of where I was so if I fell below the benchmark I’d probably feel pretty bad about it and I’d want to increase it.

SAR Non adopter P012

Our research team consulted the Cancer Care Ontario privacy team to understand which data level we could report in the email (eg, Ontario vs individual) and whether we could embed performance data in the content of the email. Performance data are considered personal information, and an error in sending could result in the disclosure of personal information of a physician to an unintended recipient. Given this potential privacy breach, Cancer Care Ontario refrains from sharing any personal information in emails. Thus, content with personal data was not an option. However, reporting aggregate data at the Ontario, regional, or Local Health Integration Network (LHIN)-level was deemed appropriate because it could not be traced back to the performance of an individual physician. Our team decided that LHIN-level data would be the most compelling solution because it was the smallest unit of aggregate data that we could report. We attempted to make the concept about comparison data more salient by associating the use of the SAR for comparison data with social norms: “Thousands of Ontario family doctors access the SAR to compare their screening rates to other family doctors in Ontario and their region.”

#### Cocreation Methods Versus Study Objectives

Our research team has extensive knowledge of behavior change theory and its application; however, at the beginning of this study, we had limited understanding of users’ experience with the SAR and what email content would compel physicians to access the SAR. Accordingly, the research team made great efforts to create conditions for meaningful and substantial user participation in the development of emails. Adopter-generated emails developed during the first workshop yielded descriptions of the SAR and meaningful content about its benefits based on user experiences. However, most content did not explicitly align with a BCT and content that did was often limited to the material incentives BCT; for example, each adopter-generated email referenced how the SAR helps to track or achieve specified levels of preventive care needed to earn their annual Preventive Care Bonus. Moreover, participants often cited comparator information as a major benefit of the SAR, but we could not identify a BCT that accurately reflected this benefit. The research team encountered tensions around the desire to be true to cocreation methods that prioritize user-generated content and the need to accomplish the study objectives of defining, operationalizing, and testing distinct BCTs.

To address this tension, we used user-generated emails from the first workshop with adopters to act as a starting point for email design and made distinctions between “base content” and “variable content.” Base content was user-generated content that was iteratively refined throughout the process and would remain constant in all emails tested in the experiment; for example, content about how the SAR provides information about patients that have abnormal results and require follow-up was well-received by users and was considered critical information by the research team for all emails to be tested. Variable content, on the other hand, was user-informed BCT content that was created by the research team, focus-tested among participants, and tested in the factorial randomized experiment.

Iterative content development began with an open-ended cocreative exploration of effective communication about the SAR. During this stage, adopters played a significant role in generating content, especially non-BCT-informed content. However, as the content evolved, study and organizational constraints were sometimes in conflict with user feedback in our quest to have distinct and defined BCTs for testing within an applied setting. These constraints often took precedence, transitioning the role of the participants from content generators to content informants as the project progressed. Further, the role of the research team moved from translators of user ideas and experiences to decision makers, ultimately, as illustrated in the tensions above. This transition of roles was necessary to accomplish the study’s objectives of testing clearly operationalized BCTs.

## Discussion

### Principal Findings

This study involved the practical application of scientific evidence and methods to the development of emails to promote the use of an audit and feedback tool. We encountered 3 tensions that may be relevant for others who are considering cocreation methods to develop similar communication interventions.

The conflict between users’ preferences and the broader scientific evidence regarding the potential effectiveness of a BCT highlights that what people seem to prefer and what works are not always the same. BCTs previously shown to be effective may not always elicit positive responses during cocreation or user testing. This tension is not unique to physician users because it also occurred in previous work with patient users relating to the development of mailings for people recovering from acute coronary syndrome [18]. Although users may be “experts of their experience” [19], they are not always experts in how to promote behavior change in themselves or in others [18]. It is necessary for researchers to be thoughtful when making design decisions because it may or may not be appropriate to make decisions solely based on whether user reactions to evidence-based content were positive or negative. It is the role of the researchers and designers to balance user feedback with the broader available evidence and explore the root cause of user reactions during data collection to make purposeful and informed design decisions.

Organizational constraints, including privacy policies, are a reality of health systems, which may limit the inclusion of personal performance data. Though users frequently recommended embedding personal performance data in the email, which was a finding consistent with best practices in audit and feedback design [20], privacy regulations did not permit our team to include such data in this setting. This created a situation in which users clearly indicated what they believed would help them and yet, we were unable to implement their suggestions.

Interestingly, the inclusion of individualized performance data may have expanded the scope of the email from an email intended to drive access to (and use of) an audit and feedback tool to an email that could actually be characterized as an audit and feedback intervention itself. There may be an opportunity in a different context with less stringent privacy regulations to provide some data, but not all data, to effectively bait the user to access the audit and feedback tool; for example, the email may present an overview of what kind of data the user could have access to if they were to use the tool. This may be more effective for communications with individuals who are underperformers rather than strong performers because participants noted that data indicating underperformance would motivate action, and research has shown that feedback is most effective when baseline performance is low [21]. However, because we were not able to test this, we do not know if this strategy would, in fact, be effective at driving access to the SAR or other audit and feedback tools.

Cocreation challenges may have occurred throughout this study because our objective of developing an email with multiple BCTs required substantial knowledge of BCTs. Adopters were able to generate concepts and non-BCT-informed content in the early stages of the research, but their ability to participate in the cocreation of a product with defined research variables such as BCTs was, not surprisingly, limited thereafter. This tension between design goals to develop the best product, service, or tool for a given context and scientific goals to identify or test generalizable concepts or theory has been noted in other contexts [22]. Cocreation methods that invite users to engage in problem solving may be most appropriate in implementation research when the study (and design) objectives are flexible, fluid, and potentially user-driven; for example, cocreation methods may be helpful when engaging users to provide input on the design and functionality of products, such as an educational application aimed at improving knowledge of clinical skills among nursing students [23] or a complex health information system [24]. However, researchers and design teams are likely to face challenges using cocreation methods when products require the application of specific scientific knowledge and should consider the dynamic and changing role of the users from content generators to content informants as the product, service, or tool develops.

### Limitations

There are several limitations to this study. First, although we started with purposive sampling, we eventually turned to convenience sampling to recruit physicians from Toronto and Kingston. Convenience sampling could potentially contribute to selection bias and failure to recruit physicians with diverse views on how to communicate to physicians about accessing an audit and feedback tool. Furthermore, we did not purposively recruit based on adherence to cancer screening guidelines. Future research with this target audience may consider recruiting based on performance level to understand if reactions to BCT-informed content differ between high performers, average performers, and underperformers. Second, our findings occurred during the development of email communications about a specific audit and feedback tool created to help physicians monitor the cancer screening status of their rostered patients. These findings may or may not apply to the development of products on different communication channels, to the development of products that deal with a different audit and feedback tool, or to a different audience such as specialists rather than family physicians; for example, it may be possible that physicians could spontaneously generate BCT-informed content or products in other contexts. Third, the analysis of the 2×2×2 factorial experiment is currently underway, and we do not yet know the impact of the interventions developed.

### Conclusions

As audit and feedback tools proliferate across health care systems, there will be an increasing need for effective communications that promote the access and use of such tools. Teams within organizations that are tasked with developing content might consider cocreation methods because they allow for the development of communications that are both user- and BCT-informed. This paper describes a repeatable series of steps that teams could use when pursuing design of implementation interventions, beginning with engaging users to understand what kind of content is compelling to them. Users provide honest feedback about what goes through their mind when they read the content and can provide thoughtful suggestions to inspire developers. However, teams working with users may need to consider whether and how they should balance user feedback with scientific evidence, organizational constraints, and study objectives. Thus, the role of users in decision making and that of BCTs along with the implementation context should all be considered during planning to determine whether cocreation methods would be appropriate to accomplish design objectives.

## Appendix

Multimedia Appendix 1 Full list of behaviour change techniques considered during development process and definitions from Michie et al.’s behaviour change technique taxonomy v1[11].

Multimedia Appendix 2 Operationalizations of behaviour change techniques to be tested in the 2X2X2 factorial experiment and evidence to support testing.

Multimedia Appendix 3 Final email consisting of all BCT-informed content to be tested in the 2X2X2 factorial randomized experiment.

## Abbreviations

BCT: behavior change technique
LHIN: local health integration network
SAR: Screening Activity Report
