# Supplementary material for: Designing Emails Aimed at Increasing Family Physicians’ Use of a Web-Based Audit and Feedback Tool to Improve Cancer Screening Rates: Cocreation Process
Source: JMIR Hum Factors. 2018 Sep 4;5(3):e25. doi: 10.2196/humanfactors.9875 (PMC6231866; doi:10.2196/humanfactors.9875)
Supplement: Multimedia Appendix 1 [file humanfactors_v5i3e25_app1.pdf]

Multimedia Appendix 1. Full list of behavior change techniques considered during development process and definitions from Michie et al.'s behavior change technique taxonomy v1[11].

Behavior Change Technique	Behavior Change Technique Taxonomy v1 Definition
**Anticipated Regret**	"Induce or raise awareness of expectations of future regret about non-performance of behavior."
**Information about Others' Approval**	"Provide information about what other people think about the behavior. The information clarifies whether others will like, approve, or disapprove of what the person is doing or will do."
**Material Incentive (behavior)**	"Inform that money, vouchers or other valued objects will be delivered if and only if there has been effort and/or progress in performing the behavior."
**Problem Solving**	"Analyze, or prompt the person to analyze, factors influencing the behavior and generate or select strategies that include overcoming barriers and/or increasing facilitators."
**Salience of Consequences**	"Use methods specifically designed to emphasize the consequences of performing the behavior with the aim of making them more memorable (goes beyond information about consequences)."
**Credible Source**	"Present verbal or visual communication from a credible source in favour of or against the behavior."
