# Supplementary material for: Designing Emails Aimed at Increasing Family Physicians’ Use of a Web-Based Audit and Feedback Tool to Improve Cancer Screening Rates: Cocreation Process
Source: JMIR Hum Factors. 2018 Sep 4;5(3):e25. doi: 10.2196/humanfactors.9875 (PMC6231866; doi:10.2196/humanfactors.9875)
Supplement: Multimedia Appendix 2 [file humanfactors_v5i3e25_app2.pdf]

Multimedia Appendix 2. Operationalizations of behavior change techniques to be tested in the 2X2X2 factorial experiment and evidence to support testing.

**Behavior Change Technique**	**Operationalization of Behavior Change Technique**	**Evidence for Behavior Change Technique**
Anticipated Regret	*How would you feel if a patient had a poor outcome because you missed an abnormal result?*	• Research has shown that anticipated regret influences physicians' decisions [15-17].<br><br>• Participant reactions revealed that the anticipated regret content provoked an emotional response that could prompt action. Reactions align with dual process models of behavior change that highlight the need to consider both the conscious and implicit processes that influence behavior [25].
Material Incentive (behavior)	*Logging into the SAR can help you maximize your screening rates and save time when calculating your Preventive Care Bonus.*	• Mixed results about the effectiveness of material incentives (e.g., financial rewards) on changing physician behavior [26-28].<br><br>• Participants endorsed the material incentive (behavior) BCT in their discussions about the value of using the SAR to calculate their Preventive Care Bonus, an annual bonus awarded to Ontarian physicians for maintaining specified levels of preventive care in their patients.

Behavior Change Technique	Operationalization of Behavior Change Technique	Evidence for Behavior Change Technique
Problem Solving	*We know accessing the SAR involves work for you and your staff. Here are three tips from other Ontario family doctors on how to fit using the SAR into your schedule:*<br>• *Email ONE® ID at ONEIDBusinessSupport@ehealthontario.on.ca to register a delegate with eHealth Ontario so they can check your report;*<br>• *Book calendar time right now to check your report; or*<br>• *Tackle a few patients at a time.*	• The positive effect of this BCT in behavior change interventions [29-31] suggests that this may be a promising BCT worth exploring in this context.<br>• Problem solving was operationalized to address the barriers of time and workload that were frequently reported by participants.
